# SPRY4-IT1 promotes survival of colorectal cancer cells through regulating PDK1-mediated glycolysis

**DOI:** 10.1080/19768354.2020.1784274

**Published:** 2020-06-25

**Authors:** Shengyuan Liu, Feng Huang, Qing Ye, Yangming Li, Jinhu Chen, Hong Huang

**Affiliations:** Department of Gastroenterology, Fujian Cancer Hospital, Fujian Medical University Cancer Hospital, Fuzhou, People’s Republic of China

**Keywords:** SPRY4-IT1, colorectal cancer (CRC), proliferation, glycolysis, molecular target

## Abstract

Colorectal cancer (CRC) becomes the third leading cause of cancer-related deaths worldwide recently. The prognosis of CRC is still poor in decades, and targeted therapy is still a potential effective treatment. Long non-coding RNAs (lncRNAs) could regulate series of cellular functions and developmental processes. LncRNA-SPRY4-IT1 (GenBank ID AK024556) is derived from an intron of the SPRY4 gene, which was highly expressed in melanoma cells and affected the progression of multiple types of cancers. However, the mechanism of SPRY4-IT1 in CRC progression remains unclear. Herein, we found the high level of SPRY4-IT1 in human colorectal cancer (CRC) tissues and cells, and correlated with patients’ prognosis. We further noticed that SPRY4-IT1 regulated CRC cell growth and glycolysis, and promoting PDK1 expression. Our data further confirmed that SPRY4-IT1 regulated CRC progression targeting PDK1. We therefore thought SPRY4-IT1 could serve as a promising molecular target for the treatment of CRC.

## Introduction

Colorectal cancer (CRC), one of the most common cancers in the world, is the third leading cause of cancer-related deaths worldwide each year (Numata et al. 2020). Abnormalities in some gene expression levels often lead to the development, progression, metastasis, and even resistance to chemotherapy of CRC (Ihemelandu et al. 2019). Common treatments for CRC include surgical resection and chemoradiotherapy (Li et al. 2020). However, due to the high metastasis of advanced CRC, its prognosis is still poor, and targeted therapy is still a potential effective treatment (Palm and Hoffe 2020). Therefore, it is urgent and necessary to understand the changes in gene expression level for the development of effective targeted drugs for CRC.

Long non-coding RNAs (lncRNAs) are non-coding protein RNAs with a length of more than 200 nucleotides that regulate various cellular functions and developmental processes (Batista and Chang 2013; Zhou et al. 2017; Sun et al. 2019). LncRNAs control gene expression in a variety of ways, including epigenetic modification and increased stability of microRNA (miRNA) and mRNA (Cao et al. 2019; Fan et al. 2019). Previous studies indicated that lncRNAs could mediated multiple physiological and pathological processes, and abnormal expression of lncRNAs could regulate the occurrence and metastasis of a variety of human tumors, including CRC (Xiang et al. 2014; Li et al. 2017; Yu et al. 2018; Cao et al. 2019).

Studies have shown that lncRNA-SPRY4-IT1 (GenBank ID: AK024556) is derived from an intron of the SPRY4 gene (Liu et al. 2017). Previous literature has confirmed that SPRY4-IT1 is highly expressed in melanoma cells than in melanocytes (Khaitan et al. 2011; Liu et al. 2016; Li et al. 2019). Downregulation of SPRY4-IT1 expression led to growth defects, decreased invasion and increased apoptosis in melanoma cells (Li et al. 2019). Other studies have shown that abnormal expression of lncRNA SPRY4-IT1 might lead to the production of abnormal htr-8 /SVneo trophoblast cells (Zou et al. 2013). In colorectal cancer, the expression of SPRY4-IT1 was up-regulated, suggesting a poor prognosis (Tan et al. 2017). SPRY4-IT1 promoted the malignant development of colorectal cancer by targeting epithelial-interstitial transformation (Cao et al. 2016). However, the mechanism of SPRY4-IT1 in CRC remains unclear.

Herein, we noticed that SPRY4-IT1 was upregulated in human CRC tissues and cells, and correlated with the prognosis of CRC patients. We found SPRY4-IT1 affected the growth and glycolysis of CRC via regulating the expression of PDK1. We therefore thought SPRY4-IT1 was a promising CRC molecular target.

## Materials and methods

### Patient samples and cell lines

72 human CRC tissues and normal tissue samples of patients were collected from Fujian Cancer Hospital, Fujian Medical University Cancer Hospital. These tissues were stored at −80°C before use. The study was approved by the Ethics Committee of Fujian Cancer Hospital, Fujian Medical University Cancer Hospital, and all the patients signed written informed consent. Normal colorectal cell line NCM460, CRC cell line T84, HT-29, and SW480 were all bought from the American Type Culture Collection (ATCC, Manassas, VA). NCM460 and HT-29 cell lines were maintained in RPMI-1640 culture medium (Gibco, Carlsbad, CA, USA); T84 and SW480 cells were maintained by the use of DMEM (Gibco; Carlsbad, CA, USA). All mediums were supplied with 10% fetal bovine serum (FBS) and penicillin/streptomycin (P/S) in a 37°C with 5% CO_2_.

### Cell transfection

Two types of p-LKO.1-lncRNA-SPRY4-IT1 shRNA plasmids and p-LKO.1-vector shRNA plasmids, and pcDNA3.1-PDK1 plasmids were obtained from Addgene. For shRNA experiments, SW480 cells were transfected with pLKO.1 vector expressing shRNA so that to deplete its expression (pLKO.1-shSPRY4-IT1). Transfection was conducted by lipofectamine 2000 (Invitrogen) according to manufacturer’s instructions.

### Quantitative PCR assay

Trizol reagent (Invitrogen, USA) were used for RNA extract from tissues or cells and total RNA were reverse transcribed using a Transcriptor cDNA Synthesis Kit (Takara, Japan). For amplification of indicated genes, SYBR Ex Taq kit (Takara, Kusatsu, Japan) was conducted based on the instructions of the kit. And the relative expression of detected genes were normalized to GAPDH. The primers in this assay were shown as follows: SPRY4-IT1, forward: 5ʹ-ATCCGAAGCGCAGACACAATTCA-3ʹ and reverse: 5ʹ-CCTCGATGTAGTCTATGTCATAGGA-3ʹ; GAPDH: forward: 5ʹ-CTCTGCTCCTCCTGTTCGAC, reverse: 5ʹ-ACCAAATCCGTTGACTCCGA.

### Immunoblotting

Proteins were extracted through SDS-PAGE assays and transferred onto NC membrane (Millipore Corporation, Bedford, MA, USA). NC membranes were subsequently blocked and incubated with specific primary antibodies against PDK1 (1:1000 dilution, ab110025, abcam, Cambridge, UK) and β-actin (1:5000 dilution, ab8226, abcam, Cambridge, UK) at 4°C for 12 h. After washing to remove non-specific binding, the membrane was treated with HRP-labeled secondary antibody in TBST buffer. Then the blots were visualized using an ECL kit. The relative protein levels were quantified by Image J.

### Cell viability and colony formation assay

SW480 cells were incubated in 96-well plates at 1000 cell density in 100 μL medium per well a day before the experiment. And then the cell viability capacity was detected by MTT assay. 1 mg/mL of MTT was treated and incubated at 37°C and maintained for 3 h. Then absorbance was measured through a Bio-Rad microplate reader (Richmond, CA, USA). Cell viability was displayed as a percentage of the OD_490_ value compared to the control.

For the colony formation, 1000 CRC cells were added into a 6-well culture plate. Cells were incubated to induce colony formation. Cells were fixed with PFA for 10 min at room temperature, and then stained using crystal violet (0.1%) staining solution for visualization of colonies for 20 min. And the plates were photographed. The numbers of the colony were counted manually.

### Cell glycolysis test

The glycolysis levels of SW480 cells was detected according to the glucose intake (ab136955), lactic acid production (ab83429), and cellular ATP levels (ab83355), which was measured by the corresponding kits bought from abcam company (Cambridge, UK). All experiments are conducted according to the relevant instructions.

### RIP assay

RIP assays were performed by the use of RIP Assay KIT. Approximately 10^8^ SW480 cells were cross-linked, lysed, then sonicated to shear the DNA into a range of 300–1000 bp. Subsequently the fraction was immunoprecipitated using anti-PDK1 or anti-IgG antibodies, and the complex was fully enriched using protein A Agarose. Beads were then washed. Total RNA was then purified and the quantitative PCR was performed.

### Statistics

Statistical analysis was performed by GraphPad. Data displayed were representive of at least 3 independent experiments as mean ± SEM. Statistical analysis was conducted through student’s t-test and *p* < 0.05 was considered as significant. * indicates *p* < 0.05, and ** indicates *p* < 0.01.

## Results

### LncRNA SPRY4-IT1 was upregulated in human colorectal cancer (CRC) tissues

**Figure 1.**
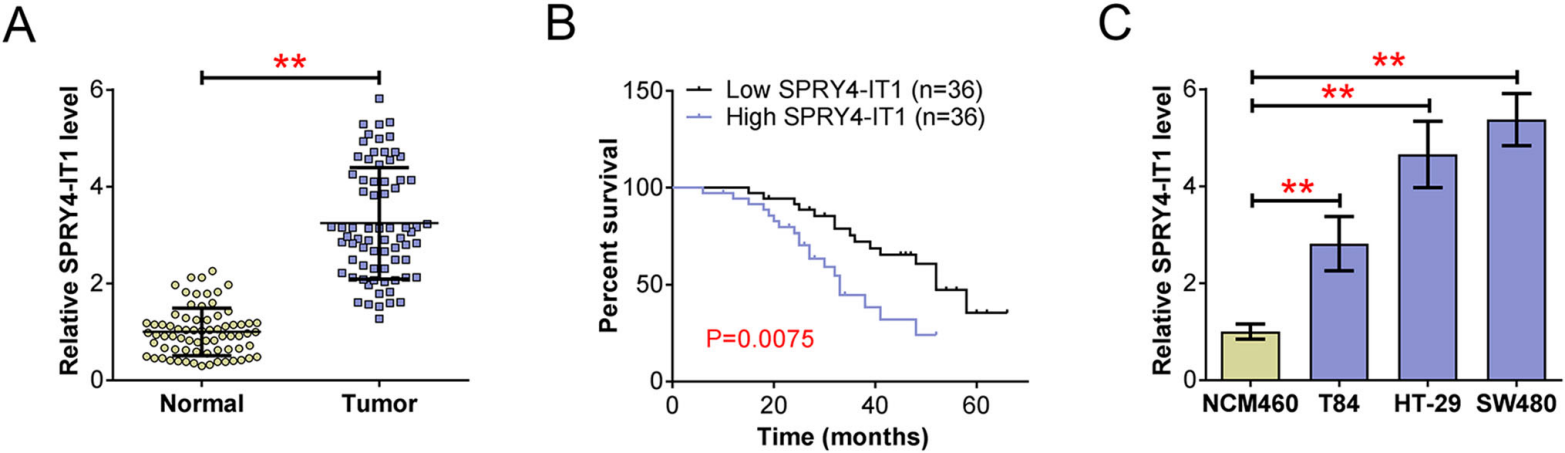
LncRNA SPRY4-IT1 was upregulated in human colorectal cancer (CRC) tissues and cells, and correlated with the prognosis of CRC patients. (A). Quantitative PCR assays showed the expression of SPRY4-IT1 in 72 CRC tissues and normal tissues. (B). Kaplan-Meier survival analysis revealed the correlation between SPRY4-IT1 expression and the survival rates of CRC patients. (C). Quantitative PCR assays revealed the expression of SPRY4-IT1 in NCM460, T84, HT-29, and SW480 cells. Results are presented as mean ± SEM, **p < 0.01.

To explore the possible effects of lncRNA SPRY4-IT1 in the progression of colorectal cancer (CRC), we first investigated the expression of SPRY4-IT1 in a total of 72 CRC tissues and normal tissues through quantitative PCR assays. Importantly, data exhibited that SPRY4-IT1 was highly expressed in CRC tissues, compared to normal tissues (Figure 1(A)). Subsequently, we investigated the effects of SPRY4-IT1 on the prognosis of CRC patients through Kaplan-Meier survival analysis. CRC patients were then divided into two groups, including SPRY4-IT1 low and high group, based on the expression level of SPRY4-IT1 in the tumor tissues. We found 36 patients showed SPRY4-IT1 high expression (50%), and the remaining exhibited low expression (50%). Notably, the data exhibited that SPRY4-IT1 expression was significantly correlated with the survival rates of CRC patients (*p* = 0.0075, Figure 1(B)), suggesting the effects of SPRY4-IT1 on CRC patients’ prognosis. We further investigated the expression of SPRY4-IT1 in human CRC cell lines, including T84, HT-29, and SW480, and normal colorectal cell line NCM460. As was expected, we found SPRY4-IT1 was also upregulated in human CRC cells, compared to the normal colorectal cells (Figure 1(C)). Our data also exhibited the higher expression of SPRY4-IT1 in SW480 cells than other CRC cells (Figure 1(C)). Therefore, we found SPRY4-IT1 was upregulated in human colorectal cancer (CRC) tissues and cells, and correlated with CRC patients’ prognosis.

### SPRY4-IT1 depletion impaired the growth and glycolysis of CRC cells *in vitro*

**Figure 2.**
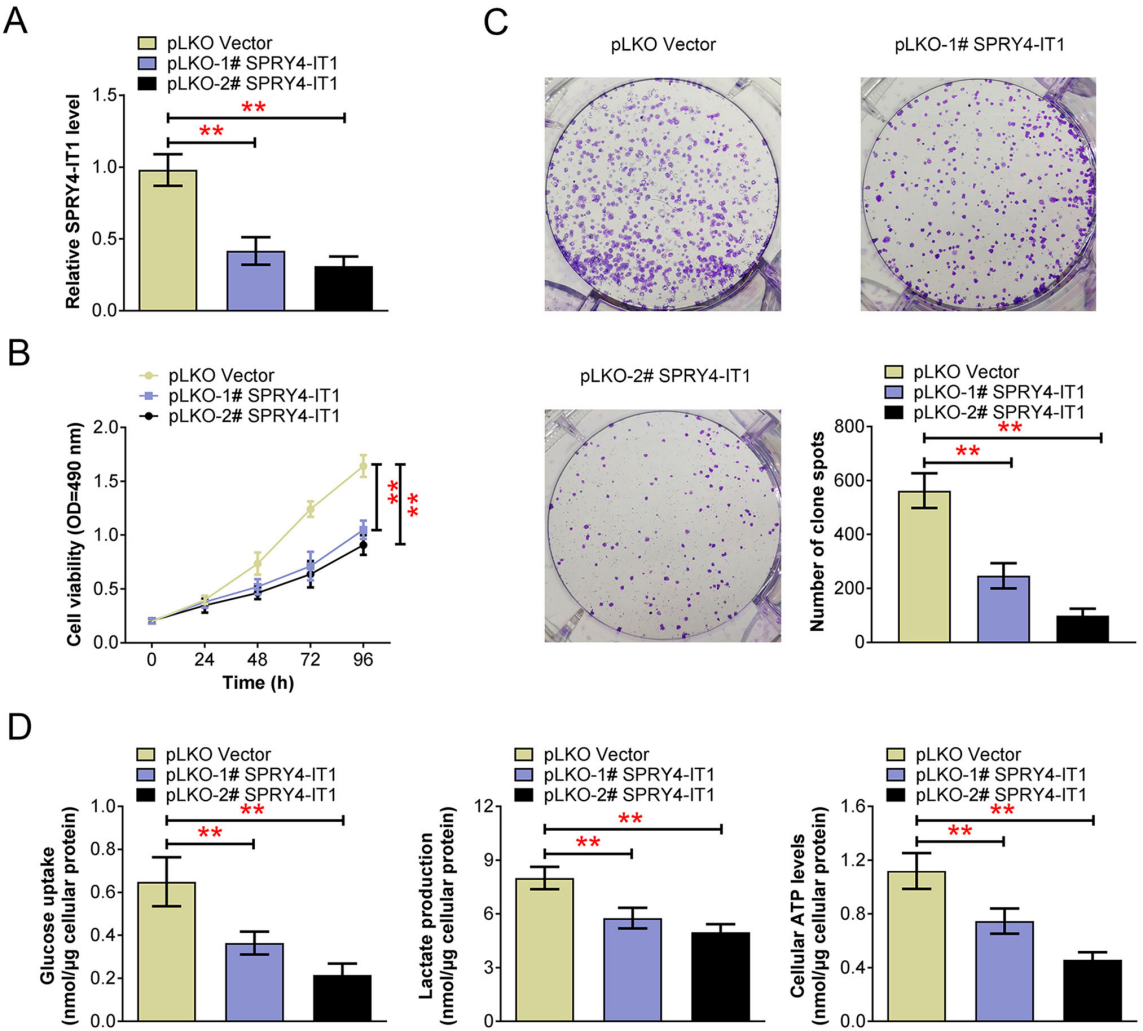
SPRY4-IT1 depletion impaired the growth and glycolysis of CRC cells *in vitro*. (A). Quantitative PCR assays showed the expression of SPRY4-IT1 after the transfection of the indicated plasmids. (B). MTT assays exhibited the change of OD_490_ levels upon the transfection of the indicated plasmids. (C). Colony formation assays were performed, and the colony number was quantified upon the transfection of the indicated plasmids in SW480 cells. (D). The levels of glucose uptake, lactate production, and cellular ATP levels were detected in SW480 cells upon the transfection of the indicated plasmids in SW480 cells. Results are presented as mean ± SEM, **p < 0.01.

Then we explore the effects of SPRY4-IT1 on CRC cells through a series of *in vitro* assays. Two pLKO shRNA plasmids targeted SPRY4-IT1 were used to deplete its expression in a type of human CRC cells, SW480 cells, in which SPRY4-IT1 was obvious high expression. Through quantitative PCR assays, we found the expression of SPRY4-IT1 was dramatically decreased upon the transfection of two types of pLKO-SPRY4-IT1 plasmids, suggesting the effective silencing (Figure 2(A)). Subsequently, we found SPRY4-IT1 ablation remarkably suppressed CRC cell viability, with the decrease OD_490_ level, which was confirmed by CCK-8 assays (Figure 2(B)). Similarly, colony formation assays confirmed the inhibition of colony formation capacity caused by the transfection of SPRY4-IT1 shRNA plasmids, with the obviously decreased colony number (Figure 2(C)).

In view of the important role of glycolysis in tumor cell growth, we then detected the levels of glucose intake, lactic acid production, and cellular ATP using the corresponding kits. As was known, these processes are important for glycolysis. Interestingly, we found that glucose intake levels were obviously decreased after SPRY4-IT1 depletion (Figure 2(D)). Additionally, lactate production levels and cellular ATP levels were also decreased upon the transfection of SPRY4-IT1 shRNA plasmids in SW480 cells (Figure 2(D)). Therefore, SPRY4-IT1 depletion impaired the growth and glycolysis of CRC cells *in vitro*.

### SPRY4-IT1 regulates the expression of PDK1 in CRC cells

**Figure 3.**
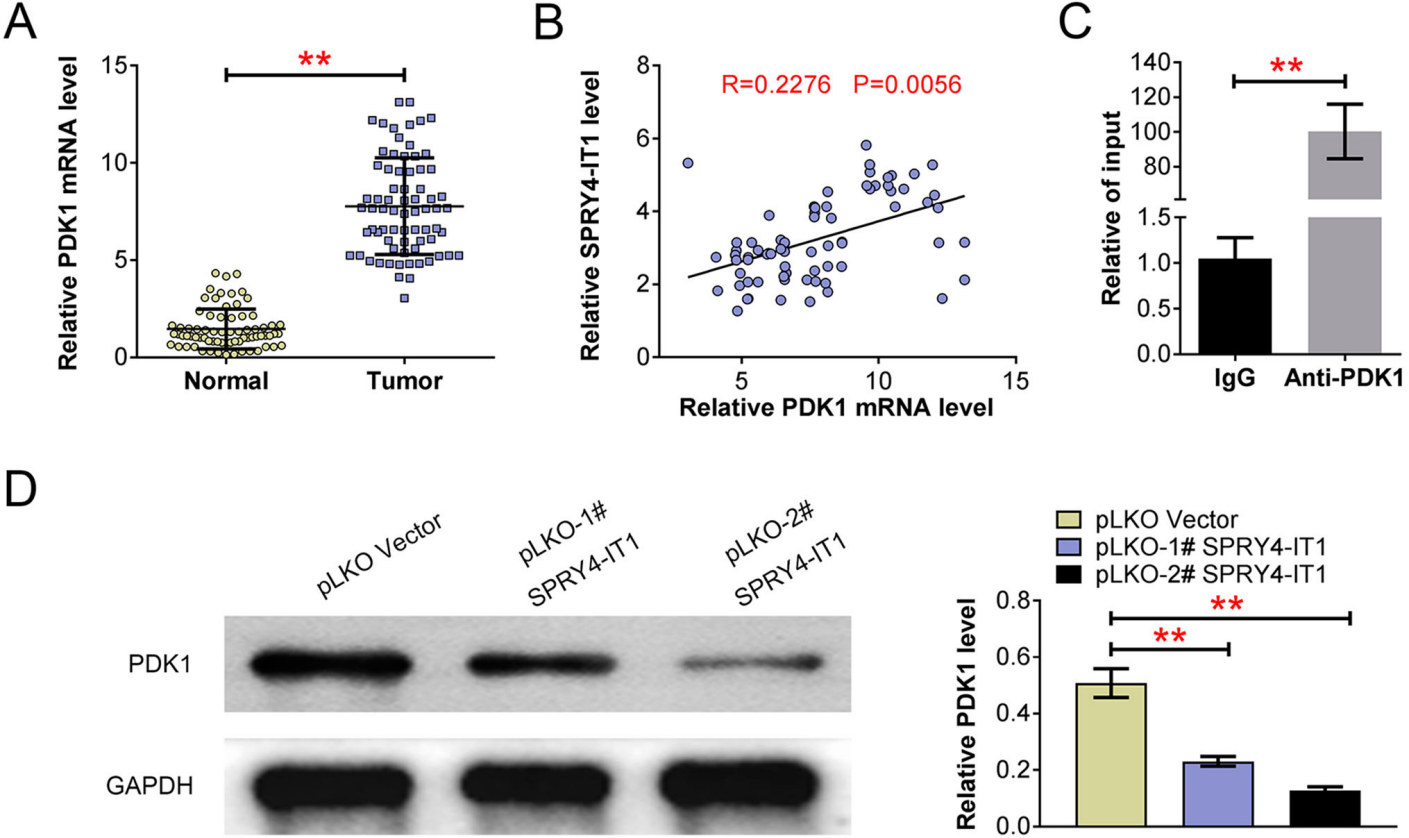
SPRY4-IT1 regulates the expression of PDK1 in CRC cells. (A). Quantitative PCR assays showed the mRNA levels of PDK1 in 72 CRC tissues and normal tissues. (B). The correlation analysis between SPRY4-IT1 and PDK1 expression in CRC tissues. (C). RIP assays exhibited the interaction between PDK1 protein and SPRY4-IT1. (D). Immunoblot assays showed the expression of PDK1 in SW480 cells after the transfection of the indicated plasmids. Results are presented as mean ± SEM, **p < 0.01.

Our previous data confirmed the effects of SPRY4-IT1 on CRC cell growth and glycolysis, and we then focused on a glycolysis regulator, PDK1, which was previously reported to be affected by SPRY4-IT1. Interestingly, we also found the high expression of PDK1 in 72 CRC tissues, compared to the normal tissues (Figure 3(A)). Meanwhile, we noticed that the expression of SPRY4-IT1 was positively correlated with PDK1 mRNA level in CRC tissues (*p*=0.0056, Figure 3(B)), suggesting the correlation between SPRY4-IT1 and PDK1. Through RIP assays, we found the antibody of PDK1 could significantly enrich SPRY4-IT1, indicating the possible interaction between PDK1 and SPRY4-IT1 (Figure 3(C)). Subsequently, we performed Immunoblot assays to confirm the regulation of SPRY4-IT1 on PDK1 expression. As was expected, SPRY4-IT1 knockdown obviously decreased the expression of PDK1 in SW480 cells (Figure 3(D)). Therefore, we thought SPRY4-IT1 could regulate the expression of PDK1 in CRC cells.

### SPRY4-IT1 regulates CRC cell growth and glycolysis via regulating PDK1 expression

**Figure 4.**
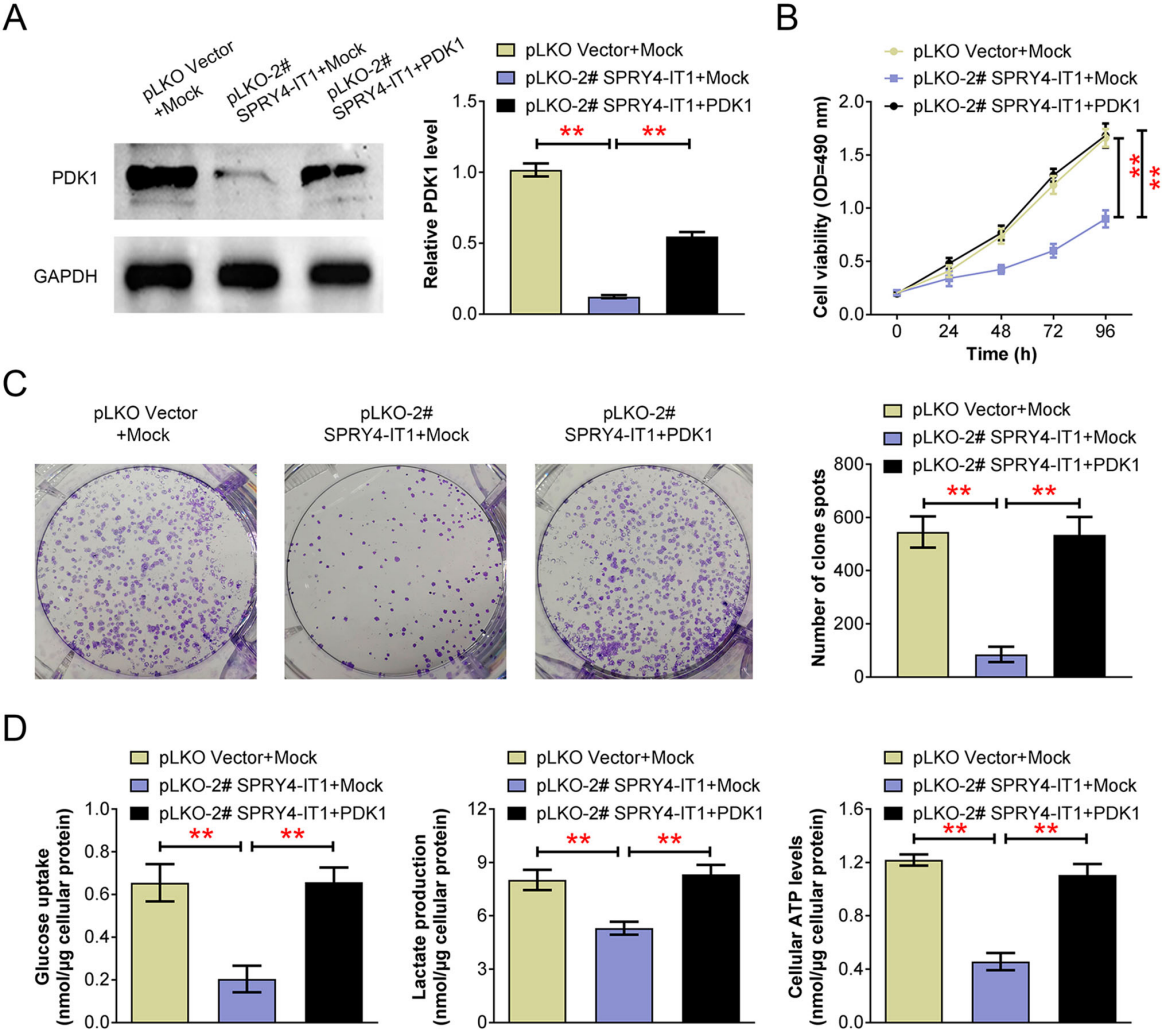
SPRY4-IT1 regulates CRC cell growth and glycolysis via regulating PDK1 expression. (A). Immunoblot assays showed the expression of PDK1 in SW480 cells transfected with the indicated plasmids. (B). MTT assays exhibited OD_490_ levels of SW480 cells upon the transfection of the indicated plasmids. (C). Colony formation assays were performed, and the colony number was quantified upon the transfection of the indicated plasmids in SW480 cells. (D). The levels of glucose uptake, lactate production, and cellular ATP levels were detected in SW480 cells upon the transfection of the indicated plasmids in SW480 cells. Results are presented as mean ± SEM, **p < 0.01.

We then detected whether SPRY4-IT1 mediated CRC cell growth and glycolysis via PDK1. Through Immunoblot assays, we noticed that the expression of PDK1 was obvious decreased after SPRY4-IT1, and PDK1 plasmid transfection could increase its expression in SPRY4-IT1-depleted SW480 cells (Figure 4(A)). Performing MTT assays, we noticed the impaired cell viability levels upon SPRY4-IT1 depletion, whereas PDK1 overexpression could obviously rescue cell viability caused by SPRY4-IT1 depletion (Figure 4(B)). Similarly, colony formation assays confirmed PDK1 overexpression significantly rescued the decrease of colony number caused by SPRY4-IT1 knockdown (Figure 4(C)). We further detected the levels of glucose intake, lactic acid production, and cellular ATP, upon the transfection of PDK1 plasmids after SPRY4-IT1 knockdown, in SW480 cells. We found that PDK1 overexpression could rescue all the decrease of glucose intake, lactic acid production, and cellular ATP levels caused by SPRY4-IT1 ablation (Figure 4(D)).

As we know, GLUT1, is a glucose transporter on the cell membrane and related to glucose metabolism. We therefore detected the expression levels of GLUT1 in control and SPRY4-IT1 depletion SW480 cells. However, no obvious different has been found in these two groups of cells (Figure S1). Therefore, we thought SPRY4-IT1 regulated glucose metabolism not through GLUT1.

Collectively, these data confirmed SPRY4-IT1 regulated CRC cell growth and glycolysis via regulating PDK1 expression.

## Discussion

For colorectal cancer (CRC), its early symptoms are not obvious, and the diagnosis is often in the advanced stage, coupled with high metastasis, increasing its treatment difficulties (Gupta et al. 2020). At present, the search for effective treatment is still imminent (Rosch et al. 2020). Targeted therapy could be regarded as a good treatment strategy for CRC, and further targeted therapy is expected to reduce the mortality of patients with advanced CRC by finding proteins or some non-coding RNAs potentially involved in regulating the development of CRC (Wang et al. 2019). In recent years, studies on lncRNAs have become increasingly abundant, and many lncRNAs are involved in regulating the progression of CRC, which may be regarded as potential therapeutic targets (Zhu et al. 2020). Fortunately, we here identified an lncRNA, SPRY4-IT1, was abnormal high expression in human CRC tissues collected in our hospital. We further found SPRY4-IT1 was correlated with the prognosis of patients with CRC, and therefore thought SPRY4-IT1 had potential to regulate CRC progression and serve as a promising CRC molecular target.

Through MTT assays and colony formation assays, we found the depletion of SPRY4-IT1 led to the impairment of proliferation in CRC cells. Furthermore, in view of the important role of glycolysis in tumor development, some studies suggested that glycolysis inhibition would affect tumor cell growth, so we examined the effect of this lncRNA on glycolysis of CRC cells. We have tested three indicators in total glucose intake, lactic acid production, and cellular ATP levels, respectively. Data showed that these indicators were obviously decreased after SPRY4-IT1 ablation, suggesting the defects in glycolysis and tumor growth. Similarly, several studies indicated the critical role of SPRY4-IT1 in cancer progression and metastasis. SPRY4-IT1 promoted the proliferation and metastasis of hepatocellular carcinoma (HCC) cells, pancreatic cancer cells, and melanoma cells (Khaitan et al. 2011; Jing et al. 2016; Guo et al. 2018; Li et al. 2019). Additionally, SPRY4-IT1 mediated the cell stemness of breast cancer cells *in vitro*, and correlated with the prognosis of breast cancer patients (Xiang et al. 2019; Song et al. 2020). Another study indicated that SPRY4-IT1 regulated the proliferation and migration of gastric cancer cells *in vitro* (Peng et al. 2015; Cao et al. 2019). These findings, together with our results in this study, confirmed the critical role of SPRY4-IT1 in cancer progression.

Importantly, we further investigated the mechanisms underlying SPRY4-IT1 promoting CRC growth and glycolysis. We found that SPRY4-IT1 could interact with PDK1, and promote the expression of PDK1, and therefore contribute to the progression of CRC. Interestingly, our data provided the evidence that SPRY4-IT1-PDK1 axis regulated cancer cell growth and metabolism. However, for different tumors, SPRY4-IT1 affects their development in many ways, often through a variety of different mechanisms. SPRY4-IT1 regulated gastric cancer progression via sponging miR-101-3p and mediating AMPK pathway (Cao et al. 2019). SPRY4-IT1 also promoted EMT of cervical cancer cells via regulating miR-101-3p/ZEB1 axis (Fan et al. 2019). In addition, SPRY4-IT1 exerted oncogenic properties via scaffolding EZH2/LSD1/DNMT1 in cholangiocarcinoma (Xu et al. 2018). Our data provide a new insight into the mechanism by which SPRY4-IT1 regulates cancer progression.

Pyruvate dehydrogenase kinase 1 (PDK1) was known as a critical regulator in cancer metabolism (Dupuy et al. 2015; Du et al. 2016). Previous study indicated that PDK1 interfered with glucose metabolism reprogramming and mitochondrial quality control in cancer (Dupuy et al. 2015). Multiple miRNAs and lncRNAs could regulate the glycolysis and mitochondrial function via targeting PDK1 (Ba et al. 2020). PDK1 could also affected the progression of multiple types of cancers, such as breast cancer, gastric cancer, and CRC (Arsenic 2014; Lian et al. 2015; Liu and Yin 2017). In this study, we found the lncRNA SPRY4-IT1 regulated CRC progression via targeting PDK1, and further suggested PDK1 could serve as a promising CRC therapeutic target. Importantly, a recent study confirmed the potential role of SPRY4-IT1 in CRC progression, and demonstrated that SPRY4-IT1 affected the proliferation and invasion of CRC cells via acting as a ceRNA of miR-101-3p, partially confirmed our findings (Jin et al. 2017).

In conclusion, we found the abnormal high expression of SPRY4-IT1 in human CRC tissues and cells, and associated with the prognosis of CRC patients. Our data further confirmed SPRY4-IT1 regulated CRC cell growth and glycolysis via regulating PDK1 expression, and therefore thought SPRY4-IT1 as a potential molecular target for CRC treatment.

## Data Availability

All data generated or analyzed during this study are included in this published article.
